# Editorial: Women in brain health and clinical neuroscience volume II: 2023

**DOI:** 10.3389/fnhum.2024.1521780

**Published:** 2024-11-21

**Authors:** Tuba Aktürk, Görsev Yener

**Affiliations:** ^1^Section Brain Stimulation and Cognition, Department of Cognitive Neuroscience, Faculty of Psychology and Neuroscience, Maastricht University, Maastricht, Netherlands; ^2^Neuroscience Research Center, Research Institute for Health Sciences and Technologies (SABITA), Istanbul Medipol University, Istanbul, Türkiye; ^3^Faculty of Medicine, Dokuz Eylül University, Izmir, Türkiye

**Keywords:** women in STEM, brain health, public health, urban pollution, neurodegeneration, older adults, default mode network, near-infrared spectroscopy (NIRS)

The underrepresentation of women in science, technology, engineering, and mathematics (STEM) fields remains a global challenge. Despite making significant contributions to scientific advancements, women constitute fewer than 30% of researchers worldwide. This disparity is perpetuated by longstanding biases and gender stereotypes that discourage girls and women from pursuing careers in STEM.

In this second volume of Women in Brain Health and Clinical Neuroscience, as women in STEM, our initiative is to showcase the research conducted by women in the field of neuroscience. This Research Topic brings together a collection of studies that not only advance our understanding of brain health but also highlight pressing public health issues affecting vulnerable populations.

The physical environment where people grow up and live, no doubt, has an inevitable effect on brain health. In the article by Harris et al., titled “*Gray space and default mode network-amygdala connectivity*”, the authors investigated the link between gray space exposure (e.g., impervious surfaces including buildings, parking lots, etc.) and amygdala-default mode network (DMN) connectivity in youth with the large sample size. Findings suggested gray space was linked with increased left amygdala-DMN connectivity, circuits that are vital in affective processing and emotion regulation-like processes. The authors concluded their results as gray space may be related to alterations in connectivity that may enhance the risk for emotion dysregulation. This research provides valuable insights into the physical built environment effect on neurodevelopmental outcomes and may help to inform future public policymakers.

Urban pollution poses a significant threat to public health, particularly concerning neurodegenerative diseases. In a comprehensive article titled “*Alzheimer and Parkinson diseases, frontotemporal lobar degeneration and amyotrophic lateral sclerosis overlapping neuropathology start in the first two decades of life in pollution exposed urbanites and brain ultrafine particulate matter and industrial nanoparticles, including Fe, Ti, Al, V, Ni, Hg, Co, Cu, Zn, Ag, Pt, Ce, La, Pr and W are key players. Metropolitan Mexico City health crisis is in progress*” Calderón-Garcidueñas, Stommel, et al. examined how exposure to ultrafine particulate matter and industrial nanoparticles contributes to the early onset of Alzheimer's, Parkinson's, frontotemporal lobar degeneration, and amyotrophic lateral sclerosis (ALS) in urban populations. Analyzing brain samples from residents of Metropolitan Mexico City, they identify a range of nanoparticles—such as Fe, Ti, Al, and others—as key players in neuropathology starting in the first two decades of life. This study highlights an ongoing health crisis and calls for immediate public health interventions to mitigate pollution exposure.

Building on this theme, in another study by Calderón-Garcidueñas, Cejudo-Ruiz, et al., titled “*Single-domain magnetic particles with motion behavior under electromagnetic AC and DC fields are a fatal cargo in Metropolitan Mexico City pediatric and young adult early Alzheimer, Parkinson, frontotemporal lobar degeneration and amyotrophic lateral sclerosis and in ALS patients*”, authors delve into the fatal role of single-domain magnetic particles with motion behavior under electromagnetic AC and DC fields. They explore how these particles act as a “fatal cargo” in pediatric and young adult patients with early neurodegenerative diseases in Metropolitan Mexico City. The research emphasizes the urgency of addressing environmental pollutants as modifiable risk factors for neurodegeneration in vulnerable populations.

Another environmental factor that might have an effect on brain health and development is malnutrition. Roger et al. investigated how early childhood malnutrition impairs adult resting brain function using near-infrared spectroscopy (NIRS) in their article titled “*Early childhood malnutrition impairs adult resting brain function using near-infrared spectroscopy*”. Their findings demonstrate significant alterations in brain activity patterns among adults who experienced malnutrition in childhood. This study underscores the importance of early nutritional interventions and adds to the growing body of evidence linking childhood health to long-term neurological outcomes.

Since the global population ages, indeed, there is a growing need for technologies that support healthy aging. In their perspective article, titled “*Advancing User-Centric Design and Technology Adoption for Aging Populations: A Multifaceted Approach*”, Stamate et al. discussed an integrative, collaborative, and multidisciplinary approach to developing and adopting assistive technologies for older adults with the showcases from authors' own experiences in the field, which makes paper's perspective even more valuable for the researchers who are working in this area. They highlight the barriers older adults face in adopting new technologies and propose strategies to improve usability and accessibility. This work is particularly relevant in the context of neurodegenerative diseases, where assistive technologies can significantly enhance the quality of life.

The articles featured in this volume not only contribute to scientific knowledge but also have significant implications for public health policy and advocacy. They collectively address how environmental factors (i.e., gray space exposure, urban pollution), early life experiences (i.e., malnutrition), and technology impact “brain health” across the lifespan. This Research Topic reinforces the necessity of interdisciplinary approaches in addressing complex health challenges, which is often overlooked in mainstream research practice.

We are proud to present this Research Topic of articles that not only advance our understanding of brain health but also exemplify the exceptional contributions of women in neuroscience. We hope that this Research Topic inspires continued efforts to promote gender equality in STEM fields and eventually all collective efforts may encourage more women to pursue careers in neuroscience research without fear of glass ceilings. As we strive to change traditional mindsets and defeat stereotypes, showcasing the work of female researchers becomes ever more critical. We extend our gratitude to all the authors, reviewers, and editors who contributed to this volume. We look forward to witnessing the future achievements of women in brain health and clinical neuroscience.

